# Trait mindful attention is associated with greater real-time emotional benefits of positive experiences: Evidence from experimental studies

**DOI:** 10.3389/fpsyt.2026.1711876

**Published:** 2026-02-05

**Authors:** Shunsuke Kuga, Masataka Ishimura, Kosuke Hagiwara, Midori Watanabe, Naoto Shibamaru, Yutaro Kawarada, Takahiro Hirai, Chong Chen, Shin Nakagawa

**Affiliations:** Division of Neuropsychiatry, Department of Neuroscience, Yamaguchi University Graduate School of Medicine, Yamaguchi, Japan

**Keywords:** aerobic exercise, consummatory pleasure, mindful attention awareness scale, mindfulness, natural environment, positive autobiographical memory, positive emotions

## Abstract

Mindfulness has been linked to improved well-being, yet most evidence relies on retrospective self-report, and it is unclear whether more mindful individuals experience greater mood enhancement when exposed to positive experiences in real time. To address this gap, we reanalyzed three experimental datasets employing task-elicited positive contexts—viewing natural images (Study 1), recalling positive memories (Study 2), and aerobic exercise (Study 3)—and conducted a mechanistic follow-up assessing pleasure sensitivity (Study 4). The results show that trait mindfulness, measured with the Mindful Attention Awareness Scale (MAAS), was positively correlated with “pleasure” in Studies 1 (r = 0.486, p = 0.014) and 2 (r = 0.478, p = 0.039). By contrast, no mindfulness-related enhancement was observed for mood responses to aerobic exercise in Study 3. In Study 1, near-infrared spectroscopy revealed a negative correlation between mindfulness and left orbitofrontal cortex activation (r = –0.488, p = 0.013). In Study 4 (n=100), higher mindfulness was associated with greater consummatory pleasure sensitivity on the Dimensional Anhedonia Rating Scale (rho = 0.307, p = 0.020). Together, these findings suggest that mindful attention may be particularly relevant for psychologically oriented or internally guided positive experiences, such as perceptual immersion or autobiographical recall, while exerting a weaker influence in physiologically driven contexts such as exercise. These results extend previous self-report and savoring measures and demonstrate experimental associations between mindfulness and enhanced mood responses to certain kinds of positive experiences.

## 1 Introduction

Mindfulness, originally rooted in Buddhist traditions, refers to intentionally attending to present-moment experience with sustained, nonjudgmental awareness (1, 2). While everyday attention often drifts towards distraction, worry, or rumination, mindfulness encourages people to remain attentive and continuously observe their sensations, thoughts, and feelings. In contemporary practice, it is widely implemented in clinical programs such as Mindfulness-Based Stress Reduction (MBSR)^2^ and has been associated with improvements in stress, pain, anxiety, and depression (3, 4). A leading mechanistic account proposes that mindfulness supports emotional well-being by strengthening interoception, the capacity to perceive and interpret internal bodily states, such as heart rate, breathing, and tension (5). Greater awareness of these physiological cues allows people to detect emotional changes early, react less intensely, and recover more quickly. This, in turn, can support disengagement from negative thoughts and re-engagement with ongoing experience.

While much mindfulness research has focused on reducing negative states, an equally important question is how mindfulness shapes responses to positive experiences. Longitudinal evidence suggests that mindful individuals are more likely to show upward affective spirals, where momentary positive emotions promote subsequent positive experiences and broaden cognition (6). Intervention studies further show that mindfulness training can increase daily positive affect and promote positive appraisal, that is, reframing stressful events in adaptive ways that preserve emotional well-being (7). One proposed behavioral mechanism is savoring, defined as intentional engagement with and appreciation of pleasant experiences, often with efforts to prolong or intensify them (8, 9). Through this kind of present-focused attention, mindfulness may facilitate engagement with pleasurable stimuli and foster stronger consummatory response.

However, despite these promising evidence, most research uses self-report questionnaires to ask participants to retrospectively describe how positive they feel or how much they savor experiences. These approaches cannot determine whether more mindful individuals actually experience greater mood enhancement when exposed to positive stimuli in real time. There is a lack of such kind of experimental evidence: very few studies have used task-elicited manipulations of positive experience followed by objective measures of emotional response.

The present study addresses this gap by examining how trait mindfulness relates to mood responses under three theoretically grounded categories of positive experience and one mechanistic follow-up. These three forms of positive experience differ in their psychological and neurobiological bases. First, contact with nature consistently improves mood and reduces stress by restoring depleted attentional resources and promoting physiological relaxation, including decreased activation of the prefrontal cortex (10, 11). Second, recalling positive autobiographical memories evokes reward-related neural states by reactivating emotionally meaningful past experiences (12). This pathway represents a distinct form of positive affect depending not on external stimuli but on personal meaning and self-relevance. Third, aerobic exercise, in contrast, is a physically embodied positive context in which mood improvement is thought to arise through neurobiological pathways, including endorphin release, dopamine modulation, and neural adaptations (13), rather than through introspection (as in recalling positive autobiographical memories) or passive sensory processing (as in exposure to natural environments). By examining these three theoretically distinct domains together, the present study explores whether associations between trait mindfulness and positive mood responses are consistent across perceptually driven, internally guided, and physiologically mediated positive experiences. We hypothesized that individuals higher in trait mindfulness would exhibit greater mood enhancement across these contexts. Mood was assessed subjectively via self-report and objectively via neuroimaging. We further hypothesized that mindfulness would relate to heightened consummatory pleasure sensitivity, which serves as an affective mechanism for mood enhancement.

To test these hypotheses, we reanalyzed three experimental datasets and conducted a new analysis. Study 1 measured mood and prefrontal activation during viewing of natural versus urban images using near-infrared spectroscopy (NIRS) (14). Study 2 examined mood changes during positive versus neutral autobiographical memory recall (15). Study 3 evaluated mood responses to an aerobic exercise intervention compared with reading (16). Study 4 assessed consummatory pleasure sensitivity using the Dimensional Anhedonia Rating Scale (DARS) (17, 18). Trait mindfulness was measured by the Mindful Attention Awareness Scale (MAAS) (19, 20), and mood by the Chen–Hagiwara Mood Test (CHAMT) (21). Here, we focus on the attentional component of mindfulness, operationalized as self-reported mindful attention versus mindlessness using the MAAS. Integrating these studies, we provide novel evidence that mindful attention is associated with the emotional benefits of positive experiences and clarify candidate mechanisms involving prefrontal regulation and reward sensitivity. Importantly, the hypothesis that individuals with higher trait mindfulness would show greater mood enhancement following positive interventions was established prior to conducting each of the three experimental studies. This hypothesis informed the inclusion of the MAAS in every experiment, even though the analytic plan was not preregistered. In contrast, the analytic approach for Study 4—examining the association between mindfulness and hedonic sensitivity—was developed in response to the findings from Studies 1–3, with the goal of elucidating the mechanisms underlying the observed effects.

## 2 Methods

### 2.1 Studies 1–3

**Figure 1:**
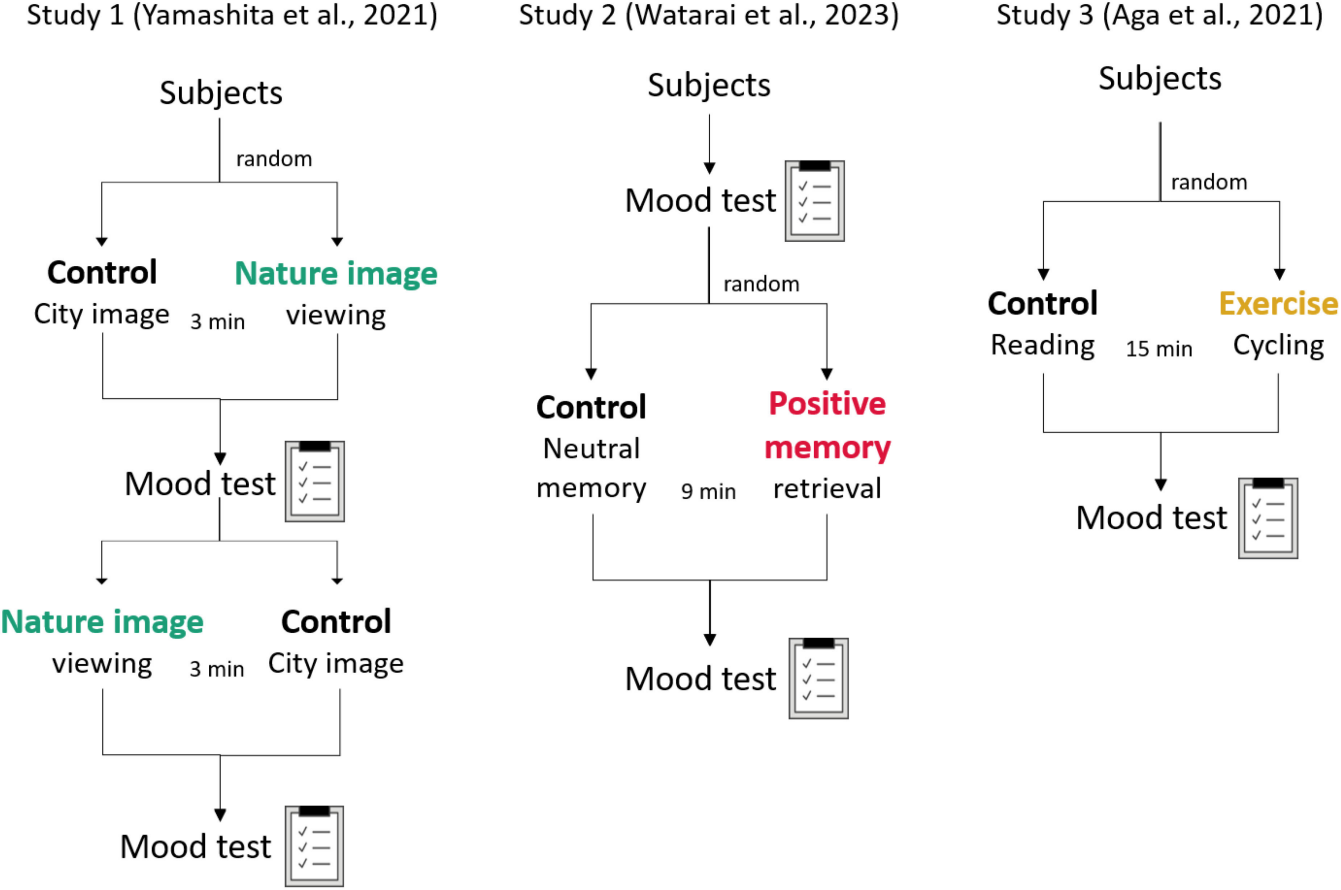
Experimental procedures of studies 1–3. The panels illustrate the natural image viewing experiment (Study 1, left), the positive memory recall experiment (Study 2, middle), and the aerobic exercise intervention (Study 3, right). Mood was assessed using the Chen–Hagiwara Mood Test (CHAMT). Study 1 employed a within-subjects crossover design (natural vs. urban images), Study 2 randomized participants to positive vs. neutral memory recall, and Study 3 randomized participants to exercise vs. reading (control).

Studies 1–3 employed comparable experimental designs in which a short intervention was followed by immediate mood assessment. In Study 1, participants viewed images of natural or urban scenes while under concurrent near-infrared spectroscopy (NIRS) (14). In Study 2, participants recalled autobiographical memories previously categorized as positive or neutral (15). In Study 3, participants performed either a 15-minute aerobic exercise session on a cycle ergometer or read quietly (16). This shared structure enabled a unified reanalysis to test the hypothesis that individuals higher in mindfulness will show greater mood enhancement from various positive experiences. The procedures of these studies are shown in **Figure 1**.

The original primary analyses of each dataset have been published independently and are cited here to clarify the distinct aims of the source studies. Study 1 tested the mood-improving effect of viewing nature images and examined associated prefrontal hemodynamic responses measured with NIRS (14). Study 2 examined how recalling positive autobiographical memories influences decision-making under risk, with mood assessed before and after recall both to quantify the affective impact of the manipulation and to evaluate whether mood changes could account for subsequent decision behavior (15). Study 3 tested the effects of acute aerobic exercise on divergent and convergent thinking, and evaluated how post-intervention mood related to these cognitive outcomes (16). In the present study, we reanalyzed these datasets with a cross-study question: whether trait mindfulness moderates immediate mood enhancement across different experimentally induced positive experiences.

Across the natural image viewing study (Study 1), the positive memory recall study (Study 2), and the exercise intervention study (Study 3), mindfulness and mood were measured using the same instruments. All studies were approved by the Institutional Review Board of Yamaguchi University Hospital and conducted following the latest guidelines of the Declaration of Helsinki. All participants provided written informed consent. Participants were recruited through campus postings, departmental website announcements, and personal referrals. They were compensated in accordance with standard university policies for research participation.

#### 2.1.1 Trait mindfulness measurement

Trait mindfulness was assessed with the Mindful Attention Awareness Scale (MAAS) (19, 20). The MAAS is a 15-item self-report scale that evaluates how frequently individuals experience inattentive or automatic behavior in daily life. Items are rated on a 6-point scale and in the raw coding, higher values indicate more frequent mindless states (i.e., lower mindfulness). For ease of interpretation, we reverse-scored all items and computed the sum so that higher scores reflect greater mindfulness (i.e., lower mindlessness). All analyses used this reverse-scored composite. Across Studies 1–3, the MAAS showed consistently strong reliability (α = 0.835, 0.876, and 0.831, respectively). The MAAS was originally chosen in these studies because it is brief, psychometrically robust (19, 20), and widely used as a dispositional measure of mindful attention in non-clinical samples, which allows a common operationalization of mindful awareness across all four datasets.

#### 2.1.2 Mood measurement

Mood was evaluated with the Chen–Hagiwara Mood Test (CHAMT) (21). Participants rated their current levels of “pleasure,” “relaxation,” and “vigor” using a 10-cm visual analog scale, scored in 1-mm increments. The CHAMT was specifically designed to overcome several drawbacks of conventional mood scales. Traditional instruments often rely on Likert-type items that assess single dimensions, require numerous responses, and may introduce response biases—such as comparisons with earlier answers or socially desirable responding. By contrast, CHAMT employs a simple, continuous scale that reduces cognitive load and minimizes these biases.

The scale is also grounded in the valence–arousal model of affect. Rather than separating valence and arousal into independent components, CHAMT captures their combined experiential quality, which is particularly relevant when examining mood-enhancing interventions. Within this framework, “relaxation” reflects positive valence with low arousal, whereas “vigor” reflects positive valence with high arousal. The use of a continuous, tick-mark-free visual analog scale further helps prevent participants from anchoring their responses to previous ratings, thereby providing a more sensitive and immediate index of momentary mood.

The CHAMT demonstrates good psychometric properties. Internal consistency is adequate, with Cronbach’s alpha values of 0.70 or higher, and same-day test–retest reliability is excellent, with intraclass correlation coefficients exceeding 0.75 (21). In terms of validity, CHAMT scores show moderate associations with established mood measures. For example, “pleasure” correlates positively with positive affect (rho = 0.265), whereas “relaxation” and “vigor” correlate negatively with state anxiety (rho = –0.423 and rho = –0.380, respectively) (21).

In Study 1, the CHAMT showed high internal consistency (Cronbach’s α = 0.885 after control images and 0.923 after nature images). In Study 2, reliability was acceptable before memory retrieval (α = 0.706) and increased after the intervention (α = 0.838 following neutral memories; α = 0.848 following positive memories). In Study 3, the CHAMT also demonstrated good reliability (α = 0.733 in the control group and α = 0.824 in the exercise group).

#### 2.1.3 Study 1: Viewing natural vs. urban images (Yamashita et al., 2021)

##### 2.1.3.1 Participants and procedure

Twenty-five healthy participants (17 men, 8 women; mean age = 23.04 ± 1.67 years) took part. In the first session, participants viewed either natural or urban images for three minutes on a high-resolution display. Immediately afterward, mood was assessed using the CHAMT. Participants then rested for three minutes. During the second session, they viewed a new set of images for three minutes, followed again by CHAMT ratings. After completing both sessions, mindfulness was assessed with the MAAS (14).

##### 2.1.3.2 Image stimuli and NIRS measurement

Participants viewed 12 images of natural environments featuring green plants in settings such as forests and meadows and 12 images of urban environments featuring buildings (control condition). All images were presented on a 27-inch high-resolution monitor (Dell S2718H, Dell Inc., Round Rock, Texas, USA). Each image was presented for 15 seconds, resulting in a total viewing time of 180 seconds. The order of natural vs. urban images was counterbalanced across participants. Both the nature and urban sets were matched in composition and each contained three images from four viewpoint categories: panoramic distant views, close-up scenes, path-oriented perspectives, and upward-angled views. All stimuli were specifically selected for this study.

**Figure 2:**
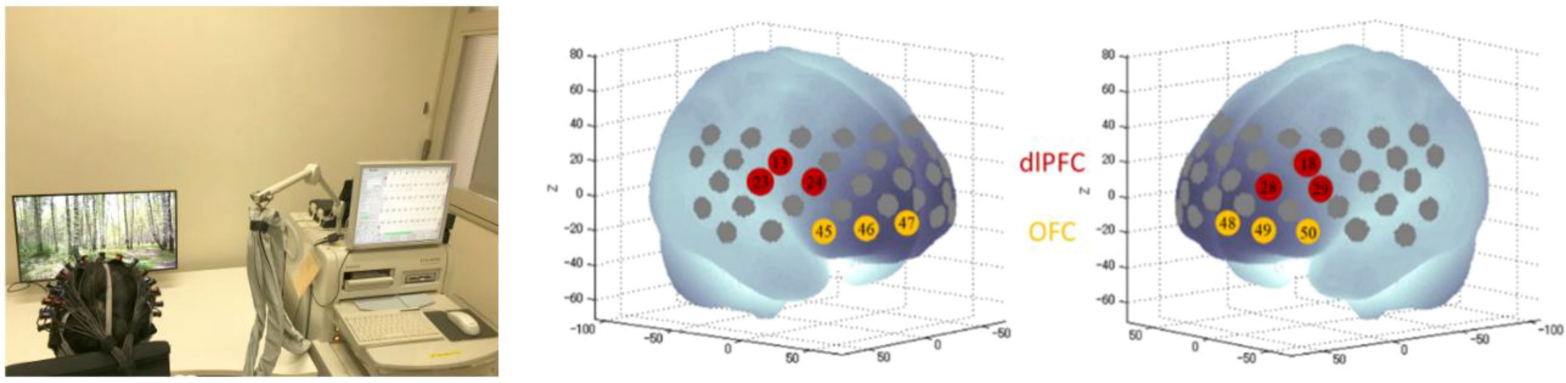
Experimental setup and NIRS measurement of oxygenated hemoglobin during image viewing (Study 1). The left panel shows a photograph of the experimental setup while a participant viewed images. The right panel illustrates the NIRS channels corresponding to the dorsolateral prefrontal cortex (dlPFC) and orbitofrontal cortex (OFC). Right panel reprinted from Yamashita et al., 2021 (14).

Brain activity was recorded using near-infrared spectroscopy (NIRS) with the ETG-4000 system (**Figure 2**), which measures changes in oxygenated hemoglobin via near-infrared light. A probe cap was placed on the scalp, allowing measurement from 52 frontal channels. The orbitofrontal cortex (OFC) and dorsolateral prefrontal cortex (dlPFC), both involved in emotional processing, were targeted. As in the initial study by Yamashita et al., 2021 (14), channels were defined as right dlPFC (13, 23, 24), left dlPFC (18, 28, 29), right OFC (45, 46, 47), and left OFC (48, 49, 50) (**Figure 2**).

#### 2.1.4 Study 2: Positive memory recall (Watarai et al., 2023)

##### 2.1.4.1 Participants and procedure

Thirty-eight undergraduates (19 men, 19 women; mean age = 21.74 ± 1.55 years) participated. Participants were randomly assigned to recall either positive or neutral autobiographical memories. Mood was assessed with the CHAMT before and after recall, and mindfulness was assessed afterward using the MAAS (15).

##### 2.1.4.2 Memory survey and recall task

On Day 1, participants completed a memory survey using an 87-item prompt list and generated at least 20 positive and 20 neutral memories. Positive memories were defined as events that evoked positive emotion both at the time of occurrence and at recall, whereas neutral memories were those that involved little or no emotional change at either time. Examples of items on the prompt list include receiving an admission or job offer, taking a family trip, receiving a birthday present, meeting a celebrity, cleaning the house, attending a remote meeting, buying mineral water from a vending machine, and inflating a bicycle tire.Within the following week, participants returned for the main experiment. They were randomly assigned to recall 20 positive or 20 neutral memories. For each memory, they were shown the original cue along with a scanned copy of their written description from Day 1 and asked to vividly reminisce for 14 seconds.

#### 2.1.5 Study 3: Aerobic exercise intervention (Aga et al., 2021)

##### 2.1.5.1 Participants and procedure

Forty participants (29 men, 11 women; mean age = 22.98 ± 1.95 years) completed the study. Participants were randomly assigned to an exercise group or a control group. Mood was assessed immediately after the intervention with CHAMT, and mindfulness was assessed afterward with the MAAS. In this study, although mood was not measured immediately before each intervention, baseline affect was assessed with the Positive and Negative Affect Schedule (PANAS). Accordingly, we included PANAS scores as covariates in our models to adjust for initial mood levels when examining the effects of mindfulness (16).

##### 2.1.5.2 Interventions

The exercise intervention consisted of 15 minutes on a cycle ergometer (Fukuda Electronics Wellbike, model BE-260), including 1 minute of warm-up, 9 minutes of exercise, and 5 minutes of rest. The control intervention involved 15 minutes of quiet reading of neutral materials unrelated to mood.

### 2.2 Study 4: pleasure sensitivity

We analyzed an additional dataset from Study 4 to test the hypothesis that individuals higher in mindfulness would show greater sensitivity to pleasure derived from positive experiences.

#### 2.2.1 Participants and procedure

One hundred undergraduate students took part (59 men, 41 women; mean age = 22.58 ± 3.40 years). They were recruited as part of an ongoing longitudinal study designed to predict later mental health outcomes (including anhedonia) using questionnaires (including the MAAS) and laboratory-based cognitive assessments. The inclusion criterion was enrollment as a regular student in a 4-year or 6-year university program. The exclusion criterion was currently receiving outpatient or inpatient treatment for depression or anxiety disorders. This study was approved by the Institutional Review Board of Yamaguchi University Hospital and conducted following the latest guidelines of the Declaration of Helsinki. All participants provided written informed consent.

After completing questions on sociodemographic characteristics, participants underwent a series of laboratory-based cognitive tasks and questionnaires—including the MAAS to assess trait mindfulness—and then completed additional mental health measures. These included the Dimensional Anhedonia Rating Scale (DARS) (17, 18). The DARS measures reward sensitivity across four categories: hobbies/leisure, food/drink, social activities, and sensory experiences. Within each category, items assess consummatory pleasure, effort, desire, and motivation. In this study, only the consummatory pleasure subscale was used as the measure of pleasure sensitivity.

### 2.3 Data analysis

Analyses were conducted in *IBM SPSS Statistics 28.0* and *Python 3.11.7*. We examined associations between trait mindfulness and (a) CHAMT mood ratings—pleasure, relaxation, and vigor—in each experiment, (b) near-infrared spectroscopy (NIRS)–based changes in oxygenated hemoglobin in Study 1, and (c) DARS pleasure-sensitivity scores in Study 4. For both Studies 2 and 3, we implemented a two-step analytical approach. First, we tested the mindfulness × condition interaction using linear regression models to determine whether the effect of mindfulness on mood differed by experimental condition. Only when the interaction term was statistically significant did we proceed to subsequent estimation of correlations between mindfulness and mood change within each condition. This approach allowed us to distinguish whether trait mindfulness specifically enhanced the effects of the intervention or was instead associated with mood changes in general regardless of intervention type. Normality was assessed with the Shapiro–Wilk test. Because DARS scores were non-normal, we used Spearman’s rank correlation; Pearson’s correlation was used for all other measures. Statistical significance was set at p <.05. Visualizations, confidence intervals, and ordinary least-squares regression were performed or estimated in *Python 3.11.7* using *Matplotlib 3.8.4*, *SciPy 1.11.4*, and *statsmodels 0.14.0*.

## 3 Results

### 3.1 Subjective mood ratings

**Figure 3:**
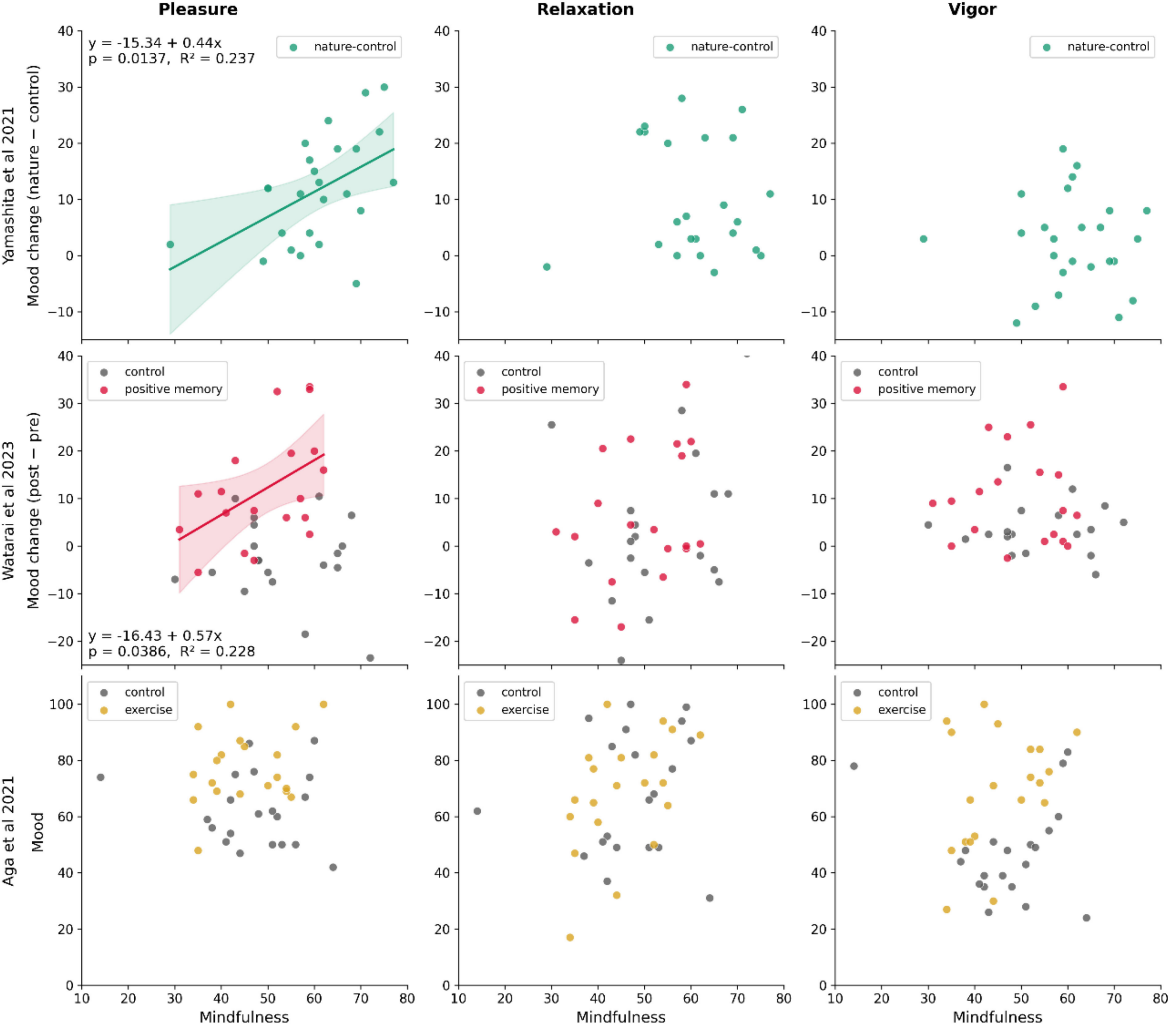
Relationship between mindfulness and mood changes across Studies 1–3. Panels show correlations between mindfulness scores (x-axis) and mood change or mood level (y-axis) for pleasure, relaxation, and vigor as assessed by the CHAMT. Study 1 (upper panel): Mood change calculated as natural minus urban image viewing (green dots). Study 2 (middle panel): Mood change calculated as post- minus pre-intervention values (gray = neutral recall, crimson = positive recall). Study 3 (lower panel): Post-intervention mood ratings are shown (gray = reading control, yellow = exercise). Whereas correlations were the primary analyses (Pearson/Spearman as appropriate), we overlay an ordinary least-squares regression line with a 95% CI to show the trend direction and precision. The regression equation, p value, and R² are shown for illustration. Significant positive associations were observed for pleasure in Studies 1 and 2.

We analyzed subjective mood ratings from the natural image viewing experiment (Study 1), the positive memory recall experiment (Study 2), and the aerobic exercise experiment (Study 3). The results are shown in **Figure 3**.

In Study 1, mindfulness was significantly correlated with ratings of pleasure (r = 0.486, 95% CI [0.113, 0.739], p = 0.014) but not relaxation (rho=-0.129, 95% CI [-0.499, 0.281], p=0.539) or vigor (r=-0.023, 95% CI [-0.414, 0.376], p=0.914, upper panel of **Figure 3**). In Study 2, linear regression revealed a statistically significant mindfulness × condition interaction for pleasure (B = 0.677, p=0.037), but not for relaxation (B = 0.0634, p=0.893) or vigor (B=-0.0519, p=0.868). Follow-up correlations conducted separately by condition showed that mindfulness was significantly associated with pleasure only in the positive memory group (r = 0.478, 95% CI [0.030, 0.766], p = 0.039) but not the control group (r = -0.134, 95% CI [-0.554, 0.341], p = 0.586). In Study 3, the mindfulness × condition interaction was not statistically significant for any of the three mood measures (B = 0.537, p= 0.231 for pleasure, B = 0.844, p= 0.240 for relaxation, and B = 0.891, p = 0.172 for vigor, respectively), even after controlling for baseline mood using PANAS scores (B = 0.455, p = 0.302 for pleasure, B = 0.725, p = 0.320 for relaxation, and B = 0.705, p = 0.273 for vigor, respectively). Overall, these findings indicate that individuals with higher mindfulness experienced greater mood enhancement from natural image viewing and positive memory recall, but not following aerobic exercise.

### 3.2 Objective neuroimaging measures

**Figure 4:**
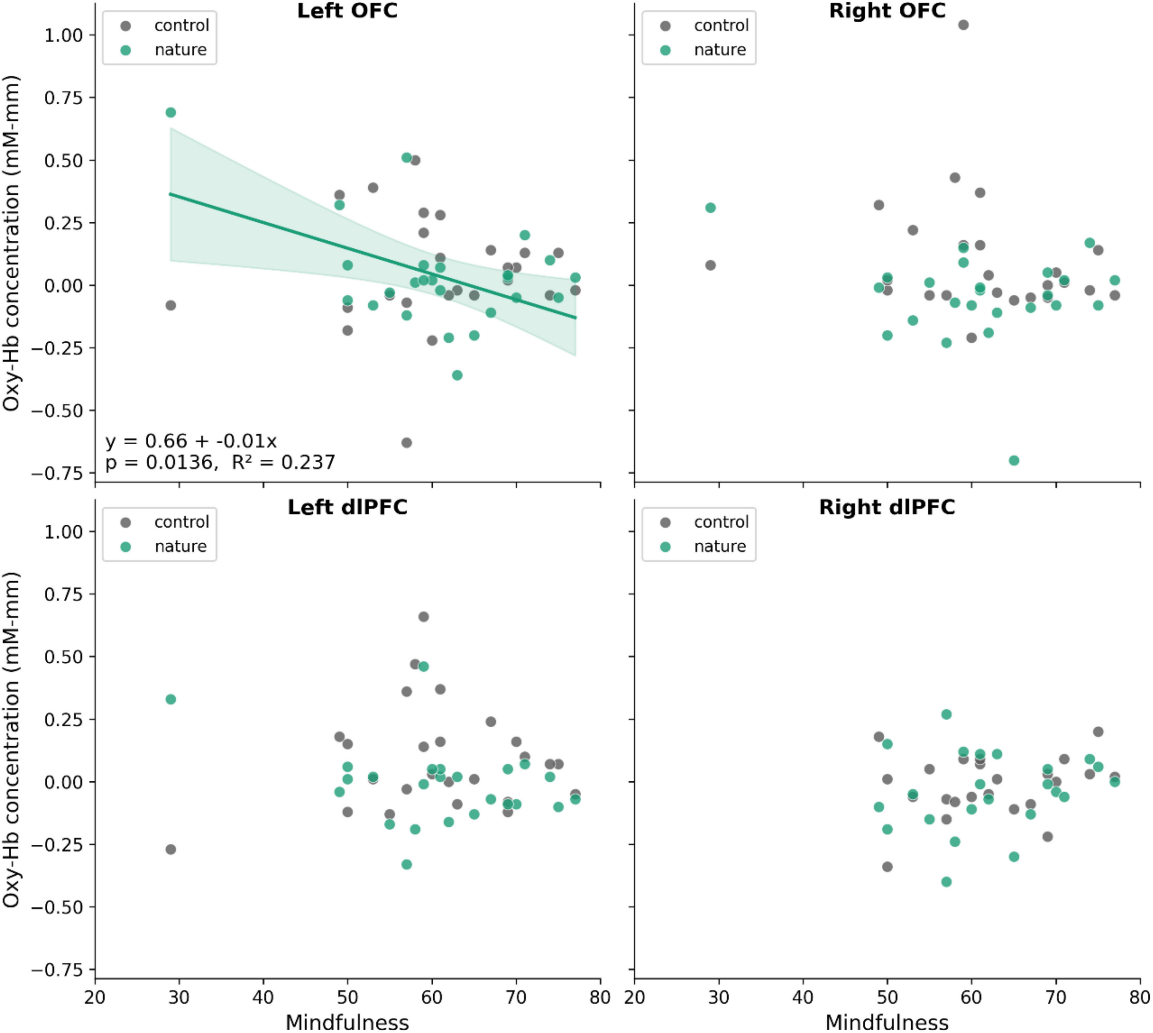
Relationship between mindfulness and oxygenated hemoglobin concentration during image viewing (Study 1). Scatterplots show mindfulness scores (x-axis) against oxygenated hemoglobin concentration (y-axis) for urban image viewing (gray) and natural image viewing (green). A regression line with 95% confidence interval is shown, together with the estimated regression equation, p value, and R². A significant negative association between mindfulness and left OFC activity was observed during natural image viewing (p < 0.05).

We next analyzed NIRS results from the natural image viewing experiment (Study 1). As shown in **Figure 4**, a significant negative correlation was found between mindfulness and oxygenated hemoglobin concentration in the left OFC while viewing natural images (r = –0.487, 95% CI [-0.740, -0.113], p = 0.014) but not control images (r = 0.059, 95% CI [-0.344, 0.444], p = 0.780). This suggests that higher mindfulness was associated with lower activation in this brain area during natural image viewing. None of the other correlations were significant (for right OFC; rho = 0.007, 95% CI [-0.398, 0.409], p = 0.975 for natural images, rho = -0.312, 95% CI [-0.636, 0.104], p = 0.137 for control images; for left dlPFC; rho = -0.128, 95% CI [-0.505, 0.291], p = 0.553 for natural images, r = 0.119, 95% CI [-0.290, 0.491], p = 0.570 for control images; for right dlPFC; r = 0.168, 95% CI [-0.262, 0.543], p = 0.442 for natural images, r = 0.226, 95% CI [-0.206, 0.584], p = 0.300 for control images).

### 3.3 Pleasure sensitivity

**Figure 5:**
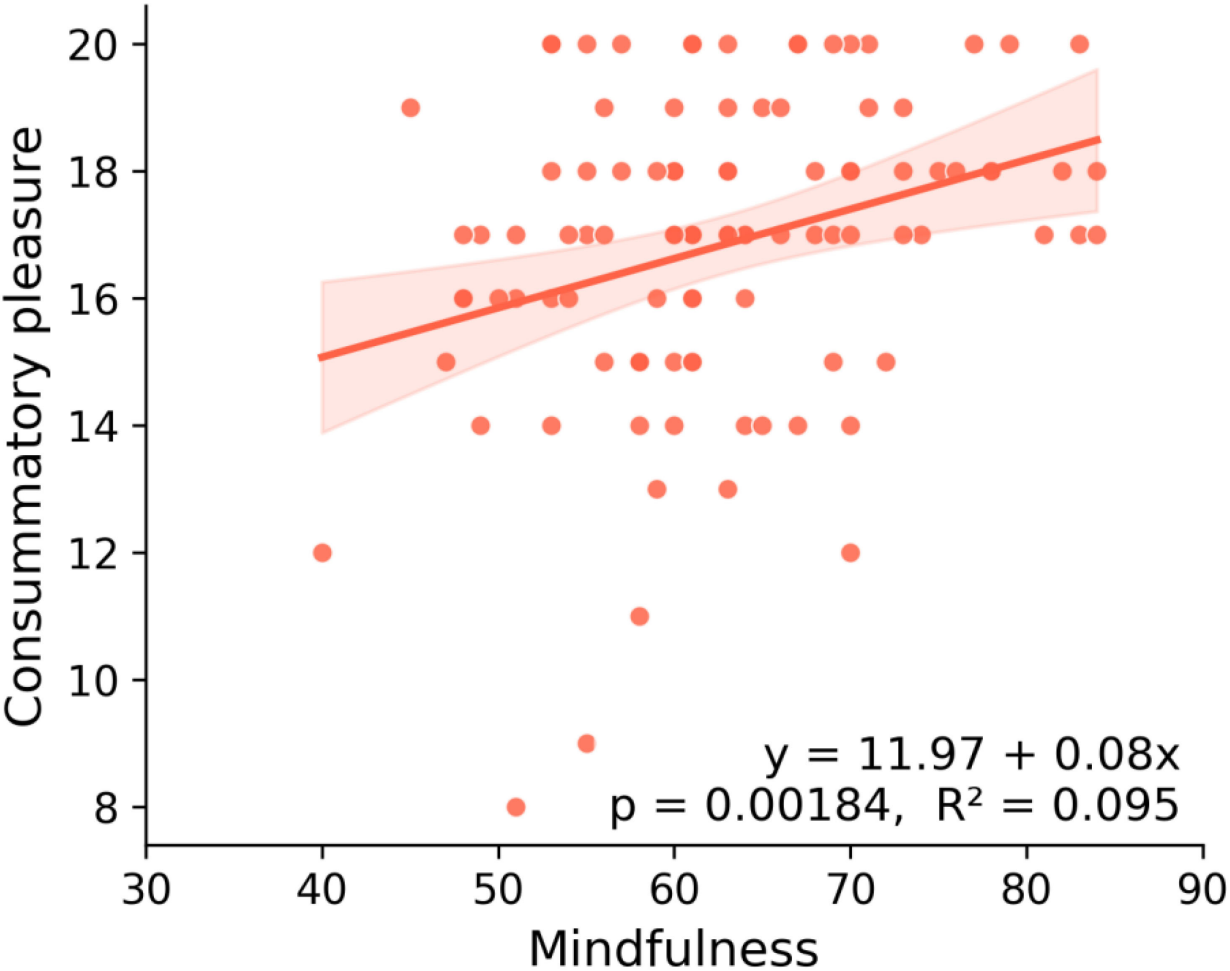
Relationship between mindfulness and pleasure sensitivity (Study 4). Scatterplot showing mindfulness scores (x-axis) and consummatory pleasure sensitivity (y-axis), as measured by the DARS. A regression line with 95% confidence interval is shown, together with the regression equation, p value, and R². A significant positive association between mindfulness and pleasure sensitivity was observed (p < 0.01).

Finally, we examined pleasure sensitivity using the DARS. The consummatory pleasure subscale score, representing the degree of enjoyment derived from positive experiences, was analyzed (**Figure 5**). A significant positive correlation was observed between mindfulness and pleasure sensitivity (rho = 0.307, 95% CI [0.118, 0.475], p = 0.020). Thus, individuals with higher mindfulness reported greater sensitivity to pleasure from positive experiences.

In addition, complementary analyses indicated that mindfulness was positively related to additional DARS dimensions, such as motivation, effort, and interest (see **Supplementary Figure S1**).

## 4 Discussion

This study has three main findings. First, across three experimental induced positive experiences (nature images, positive memory recall, aerobic exercise), trait mindfulness is associated with greater mood enhancement in two of the three contexts. Specifically, individuals higher in mindfulness experienced stronger increase in positive mood on the CHAMT pleasure subscale during natural image viewing (Study 1) and positive memory recall (Study 2), but this pattern did not extend to aerobic exercise (Study 3). Second, higher mindfulness relates to reduced left orbitofrontal cortex (OFC) activation during natural image viewing. Third, higher mindfulness is associated with greater consummatory pleasure sensitivity. Taken together, these results extend previous correlational, retrospective survey designs by demonstrating real-time mood responses to experimentally controlled positive stimuli.

### 4.1 Mindfulness and mood enhancement

Our primary contribution is to show that individuals higher in self-reported mindful attention as measured by the MAAS exhibit stronger mood gains in response to certain positive experiences. In Studies 1 and 2—viewing natural images and recalling positive memories—mindfulness significantly predicted greater increases in pleasure. This complements prior work showing that trait mindfulness predicts future positive affect (6) and that mindfulness training increases positive affect (7), which has often been interpreted through savoring (8, 9). Here, the associations are observed during experimentally controlled inductions and indexed by state mood ratings, moving beyond global self-report tendencies.

In contrast, Study 3 did not show a significant mindfulness × condition interaction, even after adjusting for baseline mood. This finding suggests that, unlike nature exposure and positive reminiscence, acute aerobic exercise did not confer greater subjective benefits for individuals higher in mindfulness. One interpretation is that the form of mindfulness assessed here—essentially mindful attention (lower mindlessness) as measured by the MAAS—may be particularly relevant in psychologically oriented or internally guided positive contexts, such as imagery, autobiographical recall, and perceptual immersion. In these contexts, attention is a key gateway for transforming sensory or cognitive content into enhanced subjective, emotional experiences (19). By contrast, physiologically driven or physically demanding contexts such as aerobic activity may rely more on autonomic and metabolic processes and less on attentional engagement, which may attenuate the relevance of mindful attention for momentary affective responses. Alternatively, ceiling effects in exercise-induced mood changes in particular pleasure, limited sample size, and individual differences in fitness levels may have obscured potential associations.

By contrast, vigor did not show a significant correlation with mindfulness, even though the original studies of the dataset that we employed reported increases in vigor following positive memory recall (15) and aerobic exercise (16). One possibility is that mindfulness is more strongly linked to low-arousal positive affect (e.g., contentment) than to high-arousal states such as vigor, which could weaken trait-level associations. Future research should test this idea by integrating physiological markers of arousal (e.g., heart rate variability, skin conductance) with self-report measures to clarify how mindfulness shapes different dimensions of positive affect.

In addition to these mechanistic considerations, it is also important to acknowledge heterogeneity in how the exercise induction is experienced. Aerobic exercise may not be universally experienced as positive or preferable. People differ markedly in how they feel about exercise, how they value physical fitness, and how much experience they have with aerobic activities. In the original Study 3 report, aerobic exercise was empirically associated with positive affective outcomes at the group level, including increases in pleasure and vigor relative to control conditions (16). Thus, here aerobic exercise is considered a physiologically mediated context in which positive mood enhancement tends to occur on average, rather than a universally enjoyable experience. Individual preference for aerobic activity was not assessed in the original study and remains an open question that future research should address.

### 4.2 Neural mechanisms in the OFC

The negative association between trait mindfulness and left OFC activation during nature viewing suggests reduced evaluative or appraisal-related engagement when the environment is intrinsically pleasant. In such contexts, lower OFC signal plausibly reflects less top-down elaboration, allowing sensory and interoceptive inputs to shape pleasant affect with fewer cognitive edits (5). This account is consistent with interpretations of nature exposure studies in which reduced prefrontal activity accompanies a relaxed, less self-referential state (11, 14, 22).

Laterality effects have varied across studies: some report bilateral reductions, others right-dominant, and others left-dominant patterns (14). In our study, the left OFC showed the strongest association with mindfulness, while the original dataset reported right OFC reductions during nature image viewing (14). Importantly, another recent study found that reduced activation in the left OFC was linked to greater feelings of pleasure in patients with anxiety disorders (23). Taken together, these findings suggest that either hemisphere may show decreased activity depending on stimulus type, participant characteristics, or clinical status.

At first glance, decreased OFC activity with higher mindfulness may seem at odds with neuroimaging work reporting increased frontal activation during meditation (24) and during processing of aversive stimuli (25). This apparent discrepancy is readily explained by task demands. In aversive or explicit regulation contexts (e.g., affect labeling), prefrontal regions—including OFC—often show increased engagement to support reappraisal or the maintenance of nonreactivity, frequently alongside dampened amygdala responses (25, 26). By contrast, in intrinsically pleasant, low-demand contexts (such as passive nature viewing) (11, 22), receptive awareness reduces the need for evaluative prediction and self-referential appraisal, yielding lower OFC signal together with higher positive affect. Directionality thus depends on whether the task calls for doing (active control) or allowing (receptive awareness).

Evidence from practice-dose studies strengthens this account. Long-term (27) or sustained (28) mindfulness practice promotes acceptance of affective states and greater emotional stability and is associated with overall medial PFC deactivation without concomitant changes in amygdala activity. This pattern fits an acceptance-based view in which mindfulness attenuates self-referential and evaluative processing rather than suppressing limbic reactivity. The present finding—reduced OFC activity during pleasant passive viewing—is consistent with this broader signature of a “quieter” frontomedial cortex under mindful acceptance.

Beyond the frontal interpretation, other components of the reward circuitry, such as the ventral striatum and dopaminergic midbrain regions (11, 29), may also be involved, as these systems play central roles in positive affect and could be differentially modulated by mindfulness. Future neuroimaging studies with fMRI should test the role of such regions.

### 4.3 Pleasure sensitivity as an affective substrate

Higher trait mindfulness was associated with greater consummatory pleasure sensitivity on the DARS (an index of hedonic capacity), which may help explain larger mood gains during positive experiences of natural image viewing and positive memory retrieval. This pattern aligns with savoring accounts (8, 9)—in which mindfulness supports attending to and amplifying positive experience. That said, it has to be noted that DARS is a self-report measure rather than a direct behavioral or physiological index. From a computational perspective, mindfulness may shift reward valuation by enhancing access to present-moment reward signals and diminishing negative biases such as rumination (22), aligning with models of positive reappraisal and interruption of maladaptive cognition (30).

This finding is also consistent with socioemotional selectivity theory, which posits that older adults focus more on present-moment experiences and consequently derive greater emotional well-being (31, 32). Although our sample was limited to students in their 20s, the observed association between mindfulness and pleasure sensitivity may represent a mechanism that operates across the lifespan. Future work should test whether this effect generalizes to older adults and clinical populations characterized by anhedonia, such as depression, where mindfulness-based interventions could potentially restore pleasure sensitivity.

However, it has to be noted that Study 4 was conducted in an independent sample and was not directly linked to the participants in Studies 1–3. Given this kind of research settings, the proposed mechanism by pleasure sensitivity here remains speculative and tentative and should not be interpreted as direct mediation or causal explanation of the mood effects observed in the experimental studies. Future research integrating longitudinal or experimental designs within the same cohort, along with formal mediation models, is needed to more rigorously test the mechanistic role of hedonic sensitivity in mindfulness-related mood enhancement.

### 4.4 Broader implications

These findings refine the role of mindfulness from a tool that buffers negative states to one that may amplify everyday positive emotion. Importantly, our results suggest that this kind of amplification may depend on the characteristics of the positive experience: mindfulness appears to strengthen affective responses in psychologically oriented or internally focused positive contexts, such as memory recall and perceptual immersion. On the other hand, the influence of mindfulness may be more limited in physiologically driven contexts, such as aerobic exercise. Practically, this pattern points to contexts in which mindful attention may naturally align with enhanced emotional responding (guided nature walks, structured positive reminiscence, brief mindful engagement with environmental cues). Our findings are also consistent with interoceptive accounts such that mindfulness may enhance access to pleasant bodily and contextual cues, which helps enable more reliable positive affect. Nonetheless, because our measures of mindfulness relied on self-report, these implications should be interpreted cautiously.

### 4.5 Limitations

An important conceptual limitation of the present study involves our operationalization of mindfulness. We used the MAAS (19, 20), which primarily assesses the frequency of mindless, inattentive states in daily life, and thus emphasizes the attentional awareness facet of mindfulness rather than nonjudgmental or accepting attitudes toward experience. Our findings therefore should be interpreted in terms of MAAS-defined mindful attention or lower mindlessness, and should not be taken as evidence about the full, multi-dimensional construct of mindfulness as articulated in broader theoretical models (1–5). Future work with multi-facet instruments or behavioral indices is required to test whether our findings generalize across different operationalizations of mindfulness.

Several additional limitations should be taken into consideration when interpreting our findings. First, although we synthesized results across three experimental datasets, the sample sizes in each study were modest, which may limit statistical power and the generalizability of the results. Second, although the positive experiences were experimentally elicited, the associations between trait mindfulness and mood responses or neural activation are individual-difference correlations, which preclude any causal inference. Third, the MAAS, which we used to assess trait mindfulness, was administered at the end of the experimental tasks. Although the MAAS is conceptualized as a trait measure and shows good temporal stability (e.g., 4-week test–retest intraclass correlation of r = 0.81; Brown & Ryan, 2003 (19)), administering it post-task raises the possibility that mood induced by the experimental manipulation may have subtly influenced participants’ self-reported mindfulness. On the other hand, administering the MAAS at the beginning of each experiment could potentially make participants more aware of their own attentional lapses and present-moment awareness, which might in turn influence how they respond to subsequent tasks. Although empirical evidence for such reactivity is limited, we wished to avoid this possible demand characteristic and therefore administered the MAAS at the end of the experimental tasks. Nonetheless, this timing choice introduces its own limitation regarding possible mood-related bias.

Fourth, although we attempted to standardize measures across studies, we did not systematically adjust or control for potential confounding factors such as baseline mood, personality traits, or lifestyle variables. For instance, Study 1 did not include a baseline mood assessment prior to the image-viewing sessions. Although the randomized within-subjects crossover design reduces concerns about initial mood differences, the absence of a pre-intervention assessment did not allow us to estimate mood changes relative to baseline and limits the interpretability of mood gains. A further limitation concerns Study 3. Although participants completed the PANAS at baseline and its scores were included as covariates to control for the confounding of baseline affective state, this measure cannot be interpreted as informing post-intervention mood, because mood was not assessed using the same instrument (CHAMT) before the intervention. Consequently, we cannot quantify mood change relative to pre-intervention levels in this study. Fifth, although we used NIRS to measure changes in oxygenated hemoglobin as an indirect indicator of neural activity, this technique has several well-known limitations, including limited spatial resolution, vulnerability to extracerebral hemodynamic influences, and the inability to capture deeper brain activities. Moreover, our discussion of reward circuitry is necessarily speculative, as it draws primarily on fMRI research and the present study did not directly assess these regions. Sixth, although examining multiple outcomes, including NIRS signals across several brain regions, provided convergent evidence, the exploratory nature of our analyses led us not to apply corrections for multiple comparisons, which may increase the risk of Type I error.

## 5 Conclusion

Across three experimental contexts, we found that higher trait mindful attention was associated with greater mood enhancement after viewing nature images and recalling positive memories, but not following aerobic exercise. Moreover, mindfulness was also associated with reduced left OFC activation while viewing nature images, and in an independent sample, with greater consummatory pleasure sensitivity. These findings suggest that trait mindfulness or more specifically mindful attention may be especially relevant for psychologically oriented or internally guided positive experiences. In other words, sustained attention which is supported by trait mindfulness may help facilitate deeper engagement with and greater enjoyment of pleasant stimuli. In contrast, physiologically driven experiences such as aerobic exercise may rely less on such attentional processes, which could explain the absence of a mindfulness-related enhancement in Study 3.

Overall, these results suggest that trait mindfulness is associated with stronger emotional responses to certain types of positive experiences in non-clinical samples. Although the positive experiences were experimentally elicited, the relationships between trait mindfulness and emotional responses reflect individual-difference associations based on modest sample sizes, and exploratory analyses with incomplete covariate control. As such, they should not be interpreted as evidence of causal mechanisms. Future preregistered, longitudinal, and experimental studies are required to clarify when and how mindful attention amplifies positive affect and to determine the pathways of such effects.

## Data Availability

The raw data supporting the conclusions of this article will be made available by the authors, without undue reservation.
