# Supplementary figures and images for: Trait mindful attention is associated with greater real-time emotional benefits of positive experiences: Evidence from experimental studies
Source: Front Psychiatry. 2026 Feb 5;17:1711876. doi: 10.3389/fpsyt.2026.1711876 (PMC12916655; doi:10.3389/fpsyt.2026.1711876)

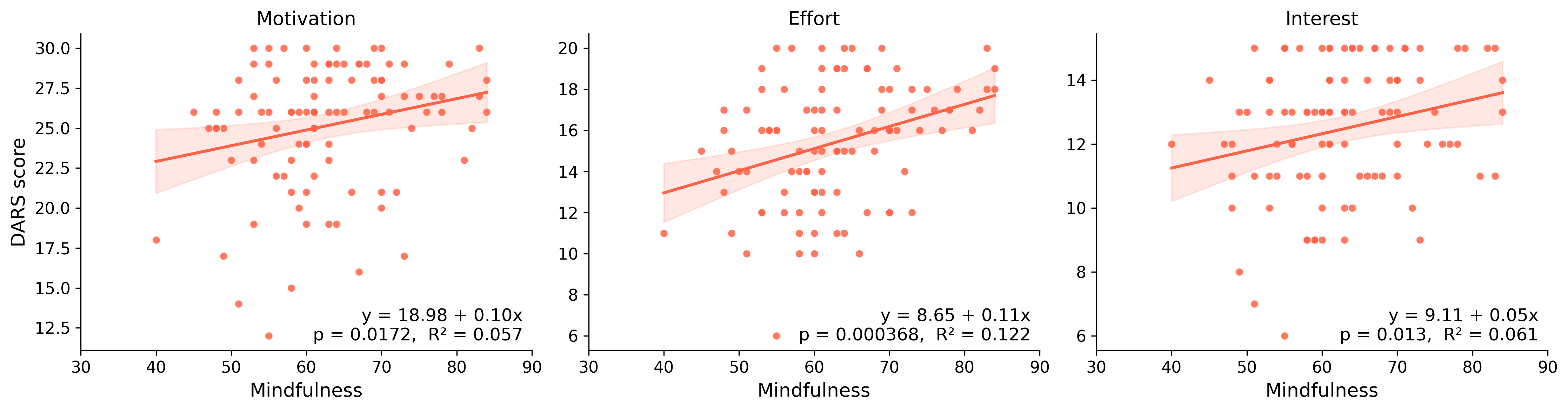

Supplement: Supplementary file 1 [file Image1.png]
